# Fostering cultural sustainability and student wellbeing: an embodied practice of teacher support in bilingual Tai Chi

**DOI:** 10.3389/fpsyg.2026.1764998

**Published:** 2026-01-23

**Authors:** Lan Li, Qianqian Fan

**Affiliations:** School of Wushu and Dance, Shenyang Sport University, Shenyang, China

**Keywords:** bilingual instruction of Tai Chi, cultural sustainability, Education for Sustainable Development, embodied learning, Self-Determination Theory, teacher support

## Abstract

**Purpose:**

Grounded in an integrated framework of Self-Determination Theory (SDT) and Embodied Cognition Theory (ECT), this study examines a bilingual Tai Chi course as a case of Education for Sustainable Development (ESD). Based on teachers’ observations and understandings of students’ sustained learning motivation and psychological wellbeing, this study explores how teachers support the satisfaction of students’ basic psychological needs through embodied interactions operating via dual pathways (physical and symbolic).

**Methods:**

Using explanatory qualitative research design, this study conducted semi-structured interviews with 16 bilingual Tai Chi teachers from four comprehensive universities in China. A three-level coding procedure (open, axial, and selective coding) was used for data analysis to inductively generate categories and core themes.

**Findings:**

The analysis identified three core practices of teacher support: cultural translation, tiered task design, and embodied collaboration. In addition, based on teachers’ descriptions of classroom experiences and their interpretations, a dual-pathway conceptual model was constructed to explicate how these instructional practices are understood by teachers as supporting students’ cultural meaning construction and the satisfaction of basic psychological needs, rather than as direct causal effects.

**Conclusion:**

This study elucidates the role of embodied instructional support in promoting cultural and social sustainability. It also contributes a theoretically grounded and practically operable framework for higher education to integrate physical and psychological wellbeing, cultural inheritance, and teaching practice within the framework of ESD.

## 1 Introduction

At the present stage, university students are facing problems such as psychological distress, emotional exhaustion, and declining learning motivation, which are jointly influenced by academic pressure and sociocultural external factors (Barbayannis et al., 2022; Al-Jayyousi et al., 2025). Within the framework of Education for Sustainable Development (ESD), psychological wellbeing is understood as an environmentally dependent outcome rather than an inherent and static trait. Psychological wellbeing is shaped jointly by individuals’ learning environments, interpersonal interactions, and adaptive teaching (UNESCO, 2020; Hansen and Barene, 2025; Norozi, 2025; Jackson et al., 2025). Traditional instructional models characterized by insufficient student autonomy and passive knowledge transmission have shown diminishing effects on long-term learning motivation and psychological wellbeing (Wang et al., 2021; Rezai et al., 2025). Existing studies indicate that simply increasing the number of courses does not improve students’ wellbeing (Bakker and Mostert, 2024; Pérez-Jorge et al., 2025), but rather, sustainable teaching strategies that integrate the emotional, cultural, and physical dimensions of learning are needed (Rodríguez-Jiménez et al., 2022; Wamsler, 2020). This study takes SDG 3 (Good Health and Wellbeing) and SDG 4 (Quality Education) of the United Nations Sustainable Development Goals (SDGs) as its intersection point, with Target 4.7—“education that promotes sustainable development and global citizenship”—as its core. Based on this, the study regards bilingual Tai Chi teacher support as a culturally distinctive instructional practice, which is not only a teaching method but also a pathway for cultivating sustainable learning motivation and culturally grounded wellbeing, both of which are core orientations emphasized in higher education within the framework of ESD (Holst et al., 2024; Buerkle et al., 2023; Ferrer-Estévez and Chalmeta, 2021).

Based on ESD-oriented teaching strategies (Wamsler, 2020; Holst et al., 2024), embodied learning provides a key pathway and emphasizes that cognition, emotion, and understanding are embedded in bodily sensations and movement experiences (Thurm et al., 2024). Taking Tai Chi as an example, movements such as “White Crane Spreads Its Wings” are commonly interpreted as embodying culturally embedded philosophical metaphors (Pedrini and Jennings, 2021). To further elaborate the contextualization of bilingual Tai Chi as an East Asian mind–body integrated cultural practice, this study drew on region-specific Chinese scholarship on Tai Chi pedagogical practices and the interpretation of movement-based cultural metaphors (Ji and Li, 2025; Xu et al., 2022). The practice within the context of bilingual Tai Chi instruction enables these cultural metaphors to be experienced, understood, and transmitted through movement, thereby providing a developmental pathway for “cultural sustainability” (Cheng and Guo, 2024). Based on Self-Determination Theory (SDT), supporting and satisfying students’ basic psychological needs is a core element in promoting their learning motivation and psychological wellbeing (Li et al., 2025). In bilingual Tai Chi classes, teacher support not only includes regular instructional guidance but also extends to: (1) providing cultural–semantic explanations of movement metaphors; (2) delivering movement guidance through embodied demonstration; and (3) offering emotional support in cross-cultural environments (Ji et al., 2025). This supportive practice, which is closely integrated with culture and the body, is a key link that connects bodily experience with basic psychological needs, thereby fostering students’ sustainable learning motivation and psychological wellbeing (Lin et al., 2022). In this study, sustainable learning motivation is defined as a motivation understanding framework oriented toward long-term maintenance and internalization, and it is characterized by three features: (1) it is described by teachers as a form of learning motivation that demonstrates continuity across time; (2) it is regarded as being associated with sustained need satisfaction and processes of meaning construction; and (3) within instructional practice, it is understood by teachers as facilitating students’ academic coping with setbacks in learning contexts, rather than manifesting as short-term situational engagement or compliance. Accordingly, this concept emphasizes how teachers, within ESD-oriented learning ecosystems, understand the maintenance and internalization of motivation, rather than making causal judgments about the motivational change process itself.

Based on existing research, this study constructs an integrated theoretical perspective: an ESD-oriented goal framework serves as the foundation, while Self-Determination Theory (SDT) and Embodied Cognition Theory (ECT) are incorporated as supplemental explanatory bases. SDT explains from the motivation pathway how teacher support provides students with sustainable learning motivation by satisfying their basic psychological needs (Zhang et al., 2023); ECT explains from the cognition–body pathway how abstract cultural concepts can be directly experienced and internalized through bodily movements and instructional demonstrations, thereby achieving cultural meaning-making (Shapiro and Stolz, 2019). Therefore, the core role of teacher support lies in designing and implementing instructional practices that can simultaneously promote embodied experience and the satisfaction of basic psychological needs, jointly pointing toward the social and cultural sustainability outcomes emphasized in ESD (Bengtsson, 2024). However, existing studies still have the following limitations: (1) contextualized practice: current research insufficiently examines how teacher support specifically operates within teaching contexts that simultaneously involve embodied, bilingual, and cultural dimensions (Jusslin et al., 2022; Liu et al., 2023); (2) theoretical integration: there is a lack of research that clearly integrates the psychological motivation mechanisms of SDT with the cultural meaning-making processes of ECT to systematically explain the emergence of sustainable learning experiences (White et al., 2021; Way and Ginns, 2024); (3) qualitative inquiry: the above mechanisms are highly contextual and process-based, yet studies relying on quantitative methods are unable to capture detailed processes such as how teachers dynamically translate cultural metaphors into understandable meanings, promote internalization through demonstration, and create a supportive atmosphere (Prananto et al., 2025; Yang et al., 2022). Therefore, an in-depth qualitative inquiry is particularly necessary.

Based on the above limitations in existing research and the need for theoretical integration, this study adopts a qualitative perspective to investigate in depth how teacher support is implemented in bilingual Tai Chi classrooms, with particular emphasis on how instructional integration through the physical–symbolic dual pathways promotes students’ sustainable learning motivation and psychological wellbeing. Accordingly, this study proposes the following research questions:

(1) What strategies do teachers adopt in bilingual Tai Chi instruction to integrate cultural metaphors and embodied demonstration?

(2) How do these instructional strategies support students’ basic psychological needs (competence, autonomy, and relatedness) within classroom interaction?

(3) How does teacher support promote students’ sustainable learning motivation and psychological wellbeing within an embodied and culturally embedded classroom practice?

## 2 Materials and methods

### 2.1 Research design and theoretical alignment

This study adopted a qualitative research design (Alhazmi and Kaufmann, 2022). Open, axial, and selective coding procedures were used as analytic tools, with the aim of developing a contextually grounded explanatory model based on teachers’ narratives, rather than generating a formal grounded theory, thereby supporting systematic interpretation. This approach is suitable for exploring “how” and “why” questions and can observe complex meanings and contextualized structures within sociocultural practices, which aligns with the requirements of research on Education for Sustainable Development for depth, process, and cultural sensitivity (Bowden, 2025).

This study is guided by an integrated framework consisting of SDT and ECT. This framework assumes that sustainable learning motivation arises from teacher support strategies and functions through dual pathways involving psychological motivation and physical–symbolic meaning construction. Therefore, the design, data collection, and analysis of this study were all aimed at observing the specific manifestations and intersections of these two dimensions within instructional interactions (Jusslin et al., 2022; White et al., 2021).

### 2.2 Participants and sampling strategy

The study adopted purposive sampling to select participants who could provide the richest and most relevant information (Campbell et al., 2020). The sample consisted of 16 bilingual Tai Chi teachers from four comprehensive universities in China. The sampling criteria were: (1) at least 1 year of bilingual Tai Chi teaching experience; (2) overseas teaching experience in institutions such as Confucius Institutes; and (3) voluntary participation with signed informed consent. In addition, the four comprehensive universities were selected because bilingual Tai Chi courses are typically offered as public physical education or elective courses, and the students enrolled in these courses represent a diverse student population. This institutional context allows comprehensive universities to move beyond the competition-oriented training logic commonly found in specialized sports universities, thereby providing a more suitable setting for examining teachers’ support patterns and instructional strategies.

The final participants included nine male and seven female teachers, with an average university teaching tenure of 4.3 years. Their professional backgrounds covered physical education (*N* = 8), English (*N* = 4), and linguistics (*N* = 4). To protect participants’ privacy, all teachers were anonymized in the analysis and identified using coded labels.

### 2.3 Data collection procedures

Data collection adopted triangulation and combined semi-structured interviews with document analysis to enhance the credibility of the study (Noble and Heale, 2019).

Semi-structured interviews: All teachers participated in individual interviews, with an average duration of 45 min. The interview outline was designed based on the integration of SDT (autonomy, competence, and relatedness needs) and ECT (embodied demonstration and cultural metaphors), in order to explore how teachers: (1) explain and translate the cultural metaphors of Tai Chi movements; (2) support student learning through demonstration and tiered tasks; and (3) construct a classroom system of emotional support and collaborative cooperation. All interviews were audio-recorded and transcribed verbatim, producing approximately 30,800 words of text. The interview protocol was designed with explicit theoretical alignment: (1) ECT-oriented questions explored how teachers translate movement-based cultural metaphors and implement a “body-first, translation-later” instructional strategy (cultural transformation and physical–symbolic balance); (2) SDT-oriented questions focused on how these practices satisfy autonomy need (choice), competence need (task stratification), and relatedness needs (collaboration). In addition, categories developed during the axial coding phase were aligned with the dual-pathway framework (see Supplementary Figure 1 and Supplementary Table 2).

Document analysis: To supplement the interview data, the study collected and analyzed the lesson plans and teaching syllabi of the participating teachers, as well as English translation materials of Tai Chi movement names. These documents provided tangible evidence for understanding teachers’ cultural translation strategies. In addition, the distribution of translation approaches corroborates the prevalence of metaphor-priority translation observed in Themes 1 and 2, while the lesson plans support the consistency between teachers’ reported practices of instructional task stratification and embodiment (see Supplementary Table 1).

### 2.4 Data analysis and coding procedures

**FIGURE 1:**
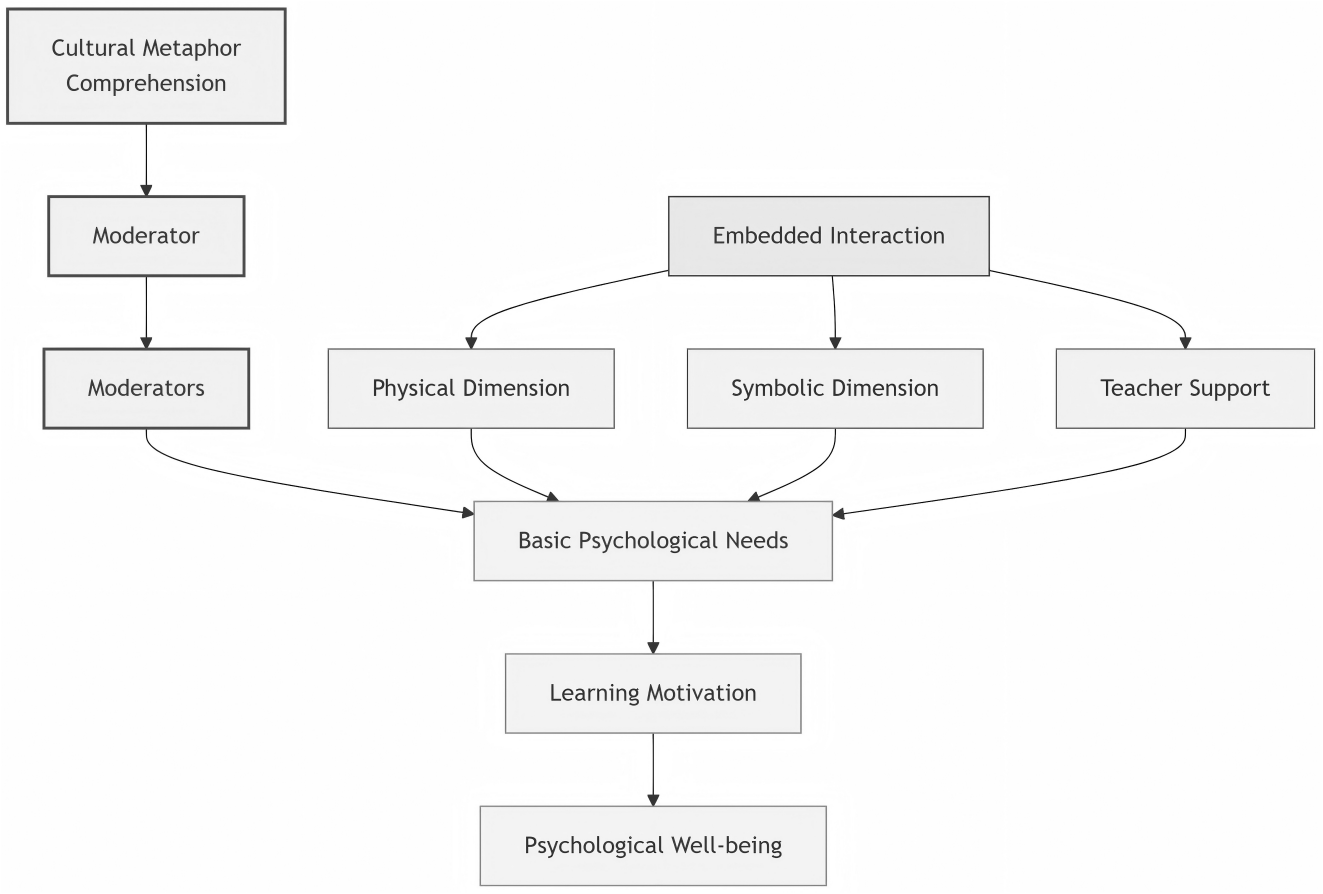
Conceptual model of embodied interaction, teacher support, and psychological wellbeing in a bilingual Tai Chi course.

Data analysis followed the principles of constructivist grounded theory and employed NVivo 12 software to conduct a systematic three-level coding process, as shown in Figure 1.

Open coding: All texts were analyzed line by line, generating 142 initial codes.

Axial coding: The large number of initial codes generated during open coding were systematically compared and categorized, and then integrated based on their conceptual properties and potential associations with the theoretical framework, forming categories.

Selective coding and theoretical integration: The core step was to integrate all categories around a “core category.” In the analytical process of this study, enjoyment, emotional safety, collaboration, and collective trust were treated as indicators of psychological wellbeing, whereas expressions related to interpersonal relationships, perceived competence, meaning, and choice were treated as manifestations of the satisfaction of autonomy, competence, and relatedness needs.

### 2.5 Trustworthiness, rigor, and ethical considerations

To ensure the rigor of the study, the following strategies were adopted: member checking (providing preliminary analyses to some participants for confirmation), two researchers independently coded 30% of the dataset, with inter-coder reliability reaching Cohen’s Kappa = 0.76, indicating a high level of consistency, as well as continuous comparison until theoretical saturation was reached (Cohen, 1960; Nowell et al., 2017). Coding discrepancies were resolved through in-depth discussions between the two researchers, with a focus on clarifying conceptual definitions and examining data excerpts until analytical consensus was reached; the revised coding decisions were then applied consistently across the entire dataset. In addition, theoretical saturation was considered to be achieved when no new codes, categories, or conceptual relationships emerged from consecutive interviews (Strauss and Corbin, 1990), a condition that was reached at the fourteenth teacher interview. Nevertheless, the researchers completed the coding of all sixteen interview transcripts and confirmed the state of saturation through a final round of constant comparative checking.

## 3 Findings

### 3.1 Overview of analytic process and emergent themes

This study generated 142 initial codes from open coding, and through axial coding, these codes were integrated into several categories. Finally, in the stage of selective coding, they were integrated into one core category: “Teacher support satisfies basic psychological needs through the physical–symbolic dual pathways, thereby supporting teachers’ observations and interpretations of students’ learning motivation and psychological wellbeing” (see Supplementary Figure 1). Around this core category, seven interrelated themes were established, which together constituted the forms of teacher support practices. In addition, to enhance analytic transparency, Supplementary Table 2 presents representative teacher descriptive texts corresponding to each theme.

To further illustrate the correspondence between the main findings and the theoretical framework, Table 1 presents three key theoretical structures and their interrelated psychological patterns.

**TABLE 1:** Integration of core theoretical chains and psychological mechanisms.

Theoretical chain	Core theme	Teacher support strategy example	Corresponding needs/motivations
Cultural transference→need satisfaction	Metaphor-priority translation	“野馬分鬃→wild horse+symbol of free spirit”	Belonging need ↑
Task differentiation → motivation internalization	Cognitive load stratification	Beginners draw circles with one hand vs. advanced learners apply complex techniques	Belonging need ↑
Embodied collaboration → wellbeing	Physical synchrony and emotional resonance	Partnered *Cloud Hands* practice enhances peer trust	Emotional need → wellbeing↑

### 3.2 Theme 1 and 2: cultural translation as embodied meaning-making

#### 3.2.1 Cultural translation of movement names (theme 1)

Teachers generally adopt a “metaphor-first” translation strategy, focusing primarily on conveying the philosophical implications of movements rather than their literal meanings. This instructional practice directly links bodily posture with cultural symbols, enabling students not only to strengthen their physical condition through movement practice but also to perceive and understand the cultural meanings embedded in the movement. This strategy corresponds to ECT, which suggests that abstract meanings are constructed through bodily experience (Shapiro and Stolz, 2019; Jusslin et al., 2022). In addition, when students understand the cultural implications of the movements, they are more likely to develop a sense of cultural identification, thereby satisfying the basic psychological needs of relatedness (sense of belonging) and autonomy (learning meaning), a finding that aligns with the perspective of SDT (Wang et al., 2021; Li et al., 2025; White et al., 2021). When translating “White Crane Spreads Its Wings,” I explained the symbolic meanings of the crane—elegance, longevity, and tranquility (PET1). In addition, PET5 stated: I place particular emphasis on the calmness and elegance symbolized by the crane, so that students can gain a deeper understanding of the cultural connotations behind the posture.

#### 3.2.2 Managing cross-cultural misinterpretation (theme 2)

In a cross-cultural context, teachers demonstrate an active awareness of cultural mediation. This strategy primarily employs positive cultural schemas, establishing an emotionally safe classroom environment, reducing students’ learning pressure and anxiety, and laying the foundation for understanding deeper cultural meanings (Barbayannis et al., 2022; Hansen and Barene, 2025; Norozi, 2025; Alhazmi and Kaufmann, 2022). Teachers emphasized the potential risks of misinterpreting cultural metaphors embedded in movement names. As one teacher explained, “To avoid misunderstanding the cultural metaphor of *Parting the Wild Horse’s Mane*, I clarify that it symbolizes freedom rather than harm to animals” (PET2). Teachers described such clarification as an important form of cultural mediation, which helps maintain emotional safety within the learning environment and supports students’ understanding of the symbolic meanings of movements. At the instructional level, adaptive strategies were also observed. For beginners, the movement *Cloud Hands* was simplified into a single-hand circular motion, whereas more proficient students participated in paired or small-group collaborative practice (PET3).

### 3.3 Theme 3 and 6: tiered task design for motivational support

#### 3.3.1 Task differentiation and support design (theme 3)

Teachers implement systematic task tiering to match students’ different ability levels. During movement instruction, teachers simplify complex movements such as the *Cloud Hands* series into easier forms suitable for beginners to practice, whereas for more proficient learners, tasks involving paired cooperation or group collaborative creation are designed. Using task tiering to regulate cognitive load as an instructional approach ensures that all students can experience tasks that are both achievable and challenging, which directly supports students’ competence need (Wang et al., 2021; Liu et al., 2023; Yang et al., 2022). This instructional task tiering design was reflected in teachers’ descriptions. For beginners, *Cloud Hands* was simplified into a single-hand circular movement, whereas more advanced learners were encouraged to engage in group-based creation and autonomous choreography (PET3). This instructional task tiering design was described as enabling students at different proficiency levels to maintain engagement without experiencing excessive pressure or insufficient challenge (PET8).

#### 3.3.2 Motivation-based differentiated support (theme 6)

Teachers provide differentiated support based on students’ motivational levels. This strategy is consistent with motivation internalization in SDT (Wang et al., 2021; Li et al., 2025; White et al., 2021). Some teachers elaborated on this differentiated instructional approach: for students with lower learning motivation, the continuous movement *Parting the Wild Horse’s Mane* was broken down into three sequential steps; whereas for students with higher learning ability, teachers encouraged autonomous movement creation and sequence arrangement, such as aligning each trajectory step of White Crane Spreads Its Wings with its corresponding cultural meaning (ET1/ET3). Teachers understood this tiered support as a gradual approach to guiding students from externally guided participation toward autonomous participation.

### 3.4 Theme 4, 5, and 7: fostering collaboration and embodied integration

#### 3.4.1 Collaborative choreography and peer interaction (theme 4)

Previous studies have indicated that bodily mediated collaboration can significantly enhance communication among students. Such collaborative activities directly contribute to the satisfaction of students’ relatedness needs and indirectly promote psychological wellbeing through a sense of collective achievement (Barbayannis et al., 2022; Hansen and Barene, 2025; Rodríguez-Jiménez et al., 2022; Lin et al., 2022). At the practical level, teacher described collaborative choreography as a shared instructional task design process. Students worked in groups to jointly design the practice specifications and sequencing of the *Cloud Hands* movement, through which their embodied practice became more closely interconnected (PET4). At the relational level, the teacher emphasized the relational effects of such collaboration. When students jointly engage in collective movement creation, they engage in more communication and gradually build mutual trust, suggesting that these collaborative interactions generate meaningful relational outcomes (PET7).

#### 3.4.2 Physical-symbolic balance in teaching (theme 5)

In terms of instructional methods, silent bodily demonstration is generally used first, allowing students to focus on imitation and bodily experience, followed by explanations of the cultural and philosophical meanings of the movements. This instructional sequence of “body first, symbol later” is consistent with cognitive principles suggesting that body engagement contribute to reduce cognitive load, subsequently, symbolic explanation reinforces cultural identification (Shapiro and Stolz, 2019; Jusslin et al., 2022; Way and Ginns, 2024). As one teacher described, “When a new movement is practiced, I first demonstrate it silently, and then I explain the cultural meanings embedded in the movement” (PET5). Another teacher also similarly noted, “I prefer a “body first, translation later” teaching approach; when students have mastered the movement, it becomes easier for them to understand the cultural metaphors contained in it” (PET8).

#### 3.4.3 Dual-channel embodied interaction (theme 7)

Some studies indicate that the synchronized connection between language and movement can strengthen the perceptual association between linguistic and kinesthetic processes, deepen embodied understanding, and contribute to mind–body experience (Rodríguez-Jiménez et al., 2022; Jusslin et al., 2022). When students practice the Tai Chi movement *Cloud Hands* while explaining the movement steps, their language use and movement practice become more synchronized (LE2). As one teacher who required students to verbalize English phrases in ways that corresponded to the movement names during practice stated, this approach helped students memorize both the English vocabulary and the movements effectively (LE4).

### 3.5 An emergent conceptual model of teacher support

Based on the above seven themes, this study integrates a conceptual model of teacher support in bilingual Tai Chi classrooms (Figure 1). This model illustrates how specific instructional practices, operating through two theorized pathways, come to be framed by teachers as sustainability-oriented learning. The model was constructed through selective coding (see Supplementary Figure 1), with its core focus on analytically integrating teaching practices rather than treating them as isolated individual components. Each pathway is used as an analytic lens to organize recurrent instructional patterns observed in teachers’ descriptions, rather than as a measurable causal mechanism.

The model indicates that teacher support is not a single behavior but a systematic and multidimensional integrated framework. Teachers transform bodily practice into cultural experience through cultural translation, optimize competence and autonomy through tiered task design, and construct emotional connection through embodied collaborative guidance. These practices jointly function to simultaneously enhance students’ body–meaning construction process (ECT) and the satisfaction of basic psychological needs (SDT) (Shapiro and Stolz, 2019; Li et al., 2025). In addition, from the perspective of contextualized practice, the model explains how instruction contributes to the goals of Education for Sustainable Development (UNESCO, 2020; Wamsler, 2020; Buerkle et al., 2023; Tejedor et al., 2022; Ferrer-Estévez and Chalmeta, 2021).

## 4 Discussion

### 4.1 The dual-pathway mechanism: interpreting the integrated model

The findings revealed a dual-pathway structure, in which teacher support serves as the core and is associated with students’ body–cultural meaning construction and the satisfaction of basic psychological needs. These two pathways are theoretically anchored in ECT and SDT, and are further aligned with the broader goals of ESD. It is important to note that all analyses concerning students’ learning motivation, psychological wellbeing, and the satisfaction of basic psychological needs were derived from teachers’ reported observations and instructional reflections, rather than from learners’ direct self-reports. Through the three practices of cultural translation, tiered task design, and embodied collaborative guidance, teachers establish a multidimensional support system (Wang et al., 2021; White et al., 2021).

The structural core of this study lies in integration. Teachers do not merely teach cultural knowledge or correct bodily movements as isolated actions; rather, they integrate the two in instruction. The explanation of cultural metaphors in movements provides personal meaning and value, thereby satisfying students’ needs for autonomy and relatedness (Wang et al., 2021; Li et al., 2025; White et al., 2021). This integrated support approach—comprising cultural interpretation, movement demonstration, and emotional construction—creates a sustainable learning environment. It also emphasizes that in embodied, bilingual, and culturally embedded classrooms, the learning environment is a crucial foundation for shaping wellbeing and sustained learning (UNESCO, 2020; Wamsler, 2020; Holst et al., 2024; Buerkle et al., 2023; Ferrer-Estévez and Chalmeta, 2021). Therefore, the dual-pathway model proposed in this study provides a replicable explanatory framework for understanding how instructional practices simultaneously contribute to individual wellbeing (SDG 3) and the educational transmission of cultural heritage (SDG 4.7) (Tejedor et al., 2022; Thurm et al., 2024; Cheng and Guo, 2024). With regard to generalizability, the dual-pathway model proposed in this study is transferable to other embodied practices (such as yoga, Pilates, and dance) only when instructional settings similarly incorporate the following conditions: (1) a physical–symbolic interwoven process that supports meaning construction; and (2) the sustained satisfaction of autonomy, competence, and relatedness needs through structured instructional support. However, due to the culturally embedded nature of Tai Chi, the specific practice of “cultural metaphor–priority” translation may require contextual adaptation in other cultural traditions or cross-cultural classroom settings.

### 4.2 Cultural-metaphorical translation: bridging body and culturally rooted wellbeing

Cultural-metaphorical translation, as a specific form of cultural translation identified in this study, refers to teachers’ instructional practice of prioritizing culturally embedded metaphors to connect bodily movements with culturally rooted philosophical meanings. The analysis indicates that teachers’ translation behavior is a form of deep cultural translation practice. Teachers adopt a cultural metaphor–first strategy and the proactive dual-buffer strategy to avoid cultural misunderstandings, with the aim of connecting bodily postures with core concepts such as Chinese philosophical culture (see Supplementary Table 2), and this has dual effects on learners. At the ECT level, the synchronization of language and movement strengthens the association between abstract cultural imagery and concrete kinesthetic experience, promoting embodied understanding (Jusslin et al., 2022; Shapiro and Stolz, 2019). At the SDT level, when students understand the cultural metaphors of the movements, their sense of learning meaning and cultural belonging is significantly enhanced, which is the core of motivation internalization and value integration (Wang et al., 2021; White et al., 2021). From an ESD perspective, this instructional practice represents the transmission of intangible cultural heritage in higher education, enabling “cultural sustainability” to be realized through everyday embodied teaching (Cheng and Guo, 2024; Wamsler, 2020; Holst et al., 2024).

### 4.3 Tiered task design: scaffolding competence and internalizing motivation

Tiered task design is a core instructional method through which teachers support the development of students’ competence and autonomy. Teachers provide a safe starting point for beginners by using cognitive-load tiering, ensuring that they can gain a sense of experience. In addition, by granting autonomy to more proficient learners, teachers provide space for challenge and choice (Liu et al., 2023; Yang et al., 2022).

This refined form of support is consistent with the motivational internalization continuum described in SDT. For students with lower ability or motivation, the structured decomposition of tasks reduces frustration and supports their motivational internalization (White et al., 2021; Li et al., 2025; Wang et al., 2021). For students with higher ability or motivation, granting autonomy in creation and naming can effectively promote identified regulation and enhance learning motivation. Therefore, tiered design not only manages learning load but also satisfies students’ basic psychological needs by providing long-term and progressive experiences, which constitutes the core psychological foundation of sustainable learning motivation (Lin et al., 2022; Li et al., 2025). This distinction indicates that the model not only explicates how motivation is strengthened, but also explains how motivation attains sustainability through dual-channel embodied support.

### 4.4 Embodied collaboration: cultivating a climate for psychosocial wellbeing

Embodied collaborative activities are central to building an emotionally safe and supportive classroom climate that promotes students’ psychological and social wellbeing. These activities create unique contexts of nonverbal communication and emotional synchrony, in which students naturally develop a sense of trust, support, and collective achievement through bodily coordination and the joint completion of tasks (Way and Ginns, 2024; Shapiro and Stolz, 2019; Rodríguez-Jiménez et al., 2022).

Within the SDT framework, this collaboration strengthened through bodily interaction represents the satisfaction of the relatedness needs. It constructs an emotionally safe classroom environment that allows students to feel comfortable attempting new movements and expressing cultural questions (Ji et al., 2025; Wang et al., 2021; White et al., 2021). In addition, the instructional sequence adopted by teachers—bodily demonstration first, followed by cultural interpretation—effectively reduces cognitive load, making it easier for students to understand the cultural symbolic meanings based on bodily perception (Jusslin et al., 2022; Shapiro and Stolz, 2019). This social–emotional–bodily integrated classroom experience not only enhances students’ sense of enjoyment but also helps develop positive physical and mental health habits and a stable sense of community, which serves as an important foundation for higher education to promote students’ holistic wellbeing (Barbayannis et al., 2022; Norozi, 2025; Prananto et al., 2025).

### 4.5 Theoretical contributions

The main theoretical contributions of this study lie in the following three aspects: (1) Expanding the application context of SDT: SDT is extended from traditional classroom verbal interaction and structural support to embodied and culturally embedded instructional practices. This study clarifies how teachers satisfy students’ basic psychological needs through non-traditional dimensions such as “bodily demonstration,” “movement Tiering,” and “cultural interpretation”; (2) Enriching the instructional implications of ECT: The key role of teachers in embodied learning is highlighted. This study clearly demonstrates how teachers, through systematic instructional design, actively shape interactive processes to optimize the embodied construction of cultural meaning; (3) Linking practice with ESD goals: This study shows that specific courses such as bilingual Tai Chi can serve as feasible carriers for simultaneously advancing personal physical and mental wellbeing, cultural transmission, and social connection, providing an embodied practical pathway and theoretical basis for implementing ESD concepts in subject instruction.

### 4.6 Practical implications, limitations, and future directions

#### 4.6.1 Practical implications

(1) Teacher development: Training programs should strengthen components such as cultural metaphor instructional competence and cross-cultural embodied teaching strategies, helping teachers become transmitters of culture and facilitators of bodily learning.

(2) Curriculum design: It is recommended that embodied-learning courses systematically incorporate tiered tasks and collaborative activities to support students’ diverse competence development and emotional belonging needs.

(3) Goal assessment: Dimensions such as cultural understanding and sense of belonging and psychological wellbeing should be explicitly included in the instructional goals and assessment systems of embodied-learning courses, thereby promoting sustainable education in an institutionalized manner.

#### 4.6.2 Limitations and future research

This study has the following limitations that provide clear directions for future research: (1) The qualitative cross-sectional design limits the ability to capture the dynamic evolution of teacher support, embodied engagement, and psychological wellbeing; (2) The study sample was limited to bilingual Tai Chi courses in Chinese universities, which restricts the transferability to other bodily practices or cultural contexts, and relevant covariates such as language proficiency, cultural background, and embodied learning experience were not considered; (3) The cultural embeddedness of Tai Chi may limit the generalizability of the mechanisms identified in this study; and (4) although multiple strategies were adopted in this study to enhance trustworthiness, reflexive journaling was not included. Therefore, confirmability primarily relies on the transparency of the coding procedures and the availability of illustrative quotations provided in the Supplementary materials.

Future research should build upon the present study to address the aforementioned limitations: (1) Future studies should adopt longitudinal or mixed-methods designs to more comprehensively reveal or observe the causal mechanisms among teacher support, basic psychological needs satisfaction, and sustainable learning outcomes; (2) Future studies may integrate multimodal data, such as classroom video observations, student experience logs, and physiological indicators, to more comprehensively and objectively capture the micro-processes of embodied interaction; and (3) Future research should be extended to different cultural contexts, bodily practices (such as yoga, qigong, and dance), and higher education institutions across multiple regions to examine the scalability and generalizability of this study in cross-cultural and cross-program contexts.

## 5 Conclusion

This study integrates SDT and ECT to examine how teacher support operates through dual pathways of cultural and bodily interaction, and how teachers understand students’ perceived satisfaction of basic psychological needs. The findings indicate that supportive practices constructed through cultural metaphor translation, tiered instructional task design, and embodied collaborative guidance are regarded by teachers as contributing to a sustainable “mind–body–culture” integrated learning mechanism. This finding not only helps deepen the understanding of motivational and cognitive processes in embodied cultural learning, but also provides an operable theoretical and practical framework for the design of sustainable learning environments in higher education, with goals oriented toward long-term learning engagement, physical and psychological wellbeing, and cultural transmission.

## Data Availability

The original contributions presented in this study are included in this article/Supplementary material, further inquiries can be directed to the corresponding author.
