# Supplementary material for: Fostering cultural sustainability and student wellbeing: an embodied practice of teacher support in bilingual Tai Chi
Source: Front Psychol. 2026 Jan 23;17:1764998. doi: 10.3389/fpsyg.2026.1764998 (PMC12876157; doi:10.3389/fpsyg.2026.1764998)
Supplement: Supplementary file 1 [file Supplementary_file_1.docx]

**
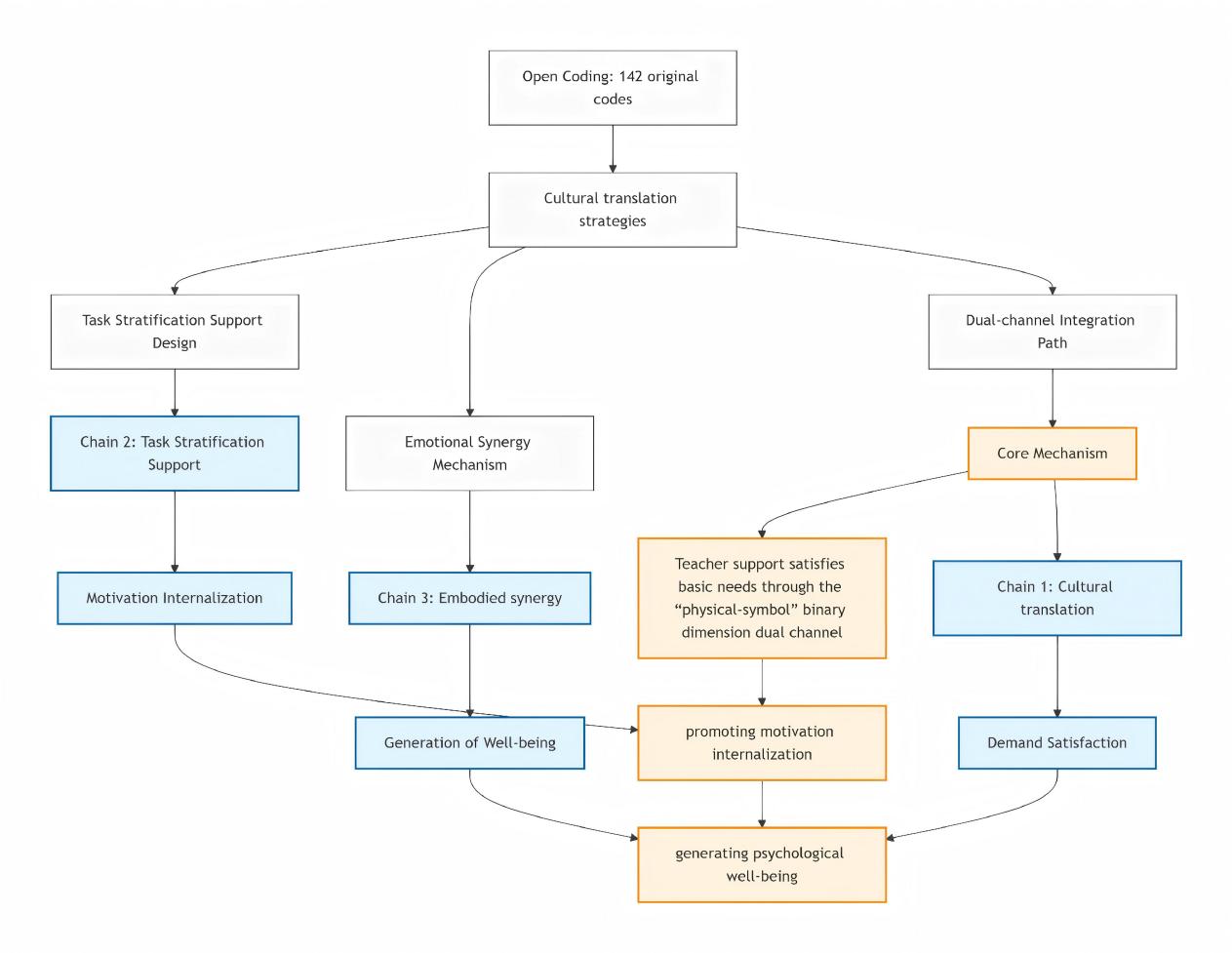
**

**Supplementary Figure 1 Theoretical Coding Framework Derived from Open Coding**

***Note:*** *Based on 142 original codes, three chains—cultural translation, task stratification, and embodied synergy—converge into a core mechanism where teacher support satisfies basic psychological needs through dual physical–symbolic pathways*

**Supplementary Table S1 Distribution of English Translation Approaches for Tai Chi Action Names**

| ***Type*** | ***Overall（%）*** | ***Example (Chinese)*** | ***Example (English)*** |
| --- | --- | --- | --- |
| Culturally Metaphorical English Expression | 45% | 左右野馬分鬃 | Parting the Wild Horse's Mane (Left and Right) |
| Free Translation | 30% | 左右摟膝拗步 | Brush Knee and Twist Step (Left and Right) |
| Literal Translation | 25% | 左右攬雀尾 | Grasp the Bird’s Tail (Left and Right) |

***Note:*** *This table summarizes the proportion of three translation approaches used in rendering 24 Tai Chi action names from Chinese into English, with representative examples for each category*

**Supplementary Table 2 Integration of Themes, Subthemes, and Illustrative Teacher Quotes**

| Core Theme | Subtheme  (Axial Coding Category) | Illustrative Teacher Quote (Representative Text) | Linked Theoretical Mechanism  (Selective Coding) |
| --- | --- | --- | --- |
| 1. Cultural Translation of Movement Names | - Metaphor-priority translation - Cultural meaning reconstruction | “When translating ‘White Crane Spreads Its Wings’, I explain the crane’s symbolic meaning—elegance, longevity, peacefulness.” (PET1/PET5/PET8) | Cultural Transference → Need Satisfaction (Autonomy & Belonging Needs ↑) |
| 2. Managing Cross-Cultural Misinterpretation | - Dual-buffer translation strategy - Symbol reinterpretation | “To avoid misunderstanding of ‘Wild Horse Parts/Shakes Its Mane’, I add that it symbolizes freedom rather than animal violence.” (PET2/PET6/PET7) | Cultural Schema Activation → Emotional Safety |
| 3. Task Differentiation and Support Design | - Cognitive load stratification - Level-based adaptation | “For beginners, Cloud Hands is simplified to one-hand circular motion; advanced students create paired variations.” (PET3/PET4/PET8) | Task Scaffolding → Competence Need Satisfaction → Motivation Internalization |
| 4. Collaborative Choreography and Peer Interaction | - Group creation mechanism - Emotional synchrony | “Students collaboratively designed the sequence of Cloud Hands, which deepened peer bonding.” (PET4/PET7) | Embodied Collaboration → Relatedness Need → Well-being |
| 5. Physical–Symbolic Balance in Teaching | - Nonverbal demonstration first - Later symbolic interpretation | “New movements are shown silently first; cultural meanings like the softness-within-strength of ‘Grasp the Bird’s Tail’ are explained afterward.” (PET5/PET8) | Physical Embodiment → Reduced Cognitive Load Symbolic Embodiment → Cultural Identity |
| 6. Motivation-Based Differentiated Support | - Support for low-motivation learners - Identity-based empowerment for high-motivation learners | “For low-motivation students, I break Wild Horse into three sub-steps; for advanced learners, I let them design variations such as a floating version of White Crane.” (ET1/ET3) | Motivation Internalization Path (from External → Identified → Intrinsic) |
| 7. Dual-Channel Embodied Interaction | - Verbal–motor synchrony - Neural coupling via instruction | “When students chant ‘Cloud Hands’ while drawing Tai Chi circles, their verbal and motor areas synchronize more effectively.” (LE2/LE4) | Embodied Interaction (Physical + Symbolic) → Flow & Well-being |

***Note:*** *PET = Physical Education Teacher; ET = Embodied Teaching Specialist; LE = Language/Embodied Integration Teacher*
